# Construction and evaluation of a column chart model and a random forest model for predicting the prognosis of hydrodistention surgery in BPS/IC patients based on preoperative CD117, P2X3R, NGF, and TrkA levels

**DOI:** 10.1186/s12911-023-02396-w

**Published:** 2023-12-14

**Authors:** Lei Pang, Zijun Ding, Hongqiang Chai, Weibing Shuang

**Affiliations:** 1 Department of Urology, Yingze District, Fifth Hospital of Shanxi Medical University (Shanxi Provincial People’s Hospital), No. 29, Shuangta East Street, Taiyuan City, 030012 Shanxi Province China; 2 Department of Neonatology, Xinghualing District, Shanxi Children’s Hospital, No. 13, Xinmin North Street, Taiyuan City, 030013 Shanxi Province China; 3 Department of Urology, Yingze District, First Hospital of Shanxi Medical University, No. 85, Jiefang South Road, Taiyuan City, 030012 Shanxi Province China; 4 First Clinical Medical College of Shanxi Medical University, No. 85, Jiefang South Road, Yingze District, Taiyuan City, 030012 Shanxi Province China

**Keywords:** Interstitial cystitis, P2X3R, Nomogram, Random forest, Prognostic model

## Abstract

**Objective:**

This study seeks to investigate independent risk factors affecting the prognoses of patients with bladder pain syndrome/interstitial cystitis (BPS/IC) following hydrodistention surgery and to develop a column chart model and a random forest model to help predict clinical outcomes.

**Method:**

A retrospective analysis was conducted on the clinical data of 1006 BPS/IC patients who visited the urology department of the Fifth Hospital of Shanxi Medical University (Shanxi Provincial People's Hospital) between June 2012 and June 2022. The patients were randomly divided into a model group (*n* = 704) and a validation group (*n* = 302). In the model group, logistic regression analysis was used to identify independent risk factors, which were used to construct a prognostic nomogram. The nomogram was evaluated by analyzing the area under the curve (AUC), calibration curve, and decision curve. These results were subsequently validated via consistency analysis (*n* = 302). And based on the random forest algorithm, we calculate the same data and construct a random forest model.

**Result:**

Multivariate logistic regression analysis revealed that age and the expression of the biomarkers CD117, P2X3R, NGF, and TrkA were independent prognostic factors for patients with BPS/IC (*P* < 0.05). Using these five indicators, a nomogram was developed to predict the risk factors for BPS/IC (scores ranged from 0 to 400). Based on the indicators, the nomogram demonstrated good prognostic performance (AUC = 0.982 and 95% confidence interva is 0.960–0.100). The correction curve indicated a high level of differentiation in the model, and the decision curve suggested positive clinical benefits. The random forest model has high accuracy and good calibration in predicting the prognosis of patients with interstitial cystitis after hydrodistention surgery.

**Conclusion:**

Age, CD117, P2X3R, NGF, and TrkA are independent prognostic factors for bladder pain syndrome/interstitial cystitis. The column chart model and random forest model constructed based on these indicators have good predictive performance for patient prognosis.

## Introduction

Bladder pain syndrome/interstitial cystitis (BPS/IC) is a chronic progressive wasting disease with multiple causes and a long course of development. The disease currently lacks a recognized definition and a clear diagnostic criteria. At present, the pathogenesis of BPS/IC is not yet clear, and it may be closely related to many pathological and physiological changes, including changes in bladder mucosal tissue, mast cell activation, autoimmune response, changes in peripheral and central nervous system nociceptors, etc. [1] Due to differences in the etiology of BPS/IC, the diagnosis and treatment of BPS/IC are also different. Currently, the diagnosis of BPS/IC is only based on the abnormal clinical manifestations of patients. However, more and more scholars have found that cystoscopy has become particularly important for the diagnosis of BPS/IC. Even so, the typical manifestation of Hunner's ulcer in BPS/IC cannot be observed in all such patients [2], Exploring the causes of BPS/IC and finding reliable diagnostic methods will be the main direction for the prevention and treatment of BPS/IC. Many scholars use random biopsy under cystoscopy to assist in the diagnosis of BPS/IC. We found an increase in mast cells in BPS/IC tissue, and mast cells can be labeled with immunohistochemistry CD117. In addition, in our previous research, we found a sharp increase in the expression of the neurogenic pain receptor P2X3R in BPS/IC tissues [3]. Other studies have shown that P2X3R plays its pathophysiological role in BPS/IC through the NGF/TrkA signaling pathway [4]. Therefore, we believe that CD117, P2X3R, NGF, and TrkA can be used to reflect the severity of BPS/IC. They are potential markers for BPS/IC and even risk factors that can affect the treatment effectiveness of BPS/IC patients. There have never been any relevant research reports on the above situation. To this end, we designed and implemented this study in order to predict independent risk factors for the prognosis of bladder pain syndrome/interstitial cystitis patients after hydrodilation surgery and establish a column chart prognosis model and a random forest model, which is of great significance in guiding clinical practice and judging the prognosis of bladder pain syndrome/interstitial cystitis patients. To this end, we constructed a clinical prediction model based on receptor scores such as CD117 and P2X3R, aiming to explore the clinical value of receptors such as CD117 and P2X3R in evaluating the prognosis of patients with interstitial cystitis.

## Materials and methods

### Research subjects

A total of 1006 patients who were diagnosed with BPS/IC and received treatment in our outpatient department between June 2012 and June 2022 were selected as the research subjects. This study was approved by the hospital's ethics committee and the batch number was assigned as (2022) Provincial Medical Lunshen Zi No.62.

The inclusion criteria were as follows: (1) patient must meet the inclusion criteria for interstitial cystitis established by AUA [5]; (2) age ≥ 25 and ≤ 65 years old; (3) availability of patient’s complete clinical data; (4) patient must have signed an informed consent form. Exclusion criteria were as follows: (1) patients with severe autoimmune diseases; (2) patients with malignant tumors; (3) patients with uncontrollable hypertension, multiple sclerosis, Parkinson's disease, spinal cord injury, cauda equina nerve injury, multisystem atrophy, or any other diseases that may affect the function of the lower urinary tract; (4) patients with severe cardiovascular and renal system diseases; (5) pregnant or lactating women; (6) patients with hematuria of unknown etiology. Based on their prognosis, patients were divided into two groups: a good prognosis group and a poor prognosis group. Each patient’s urination frequency and bladder pain level were recorded every three months.

### Data collection

Patients’ clinical and laboratory data were obtained from electronic medical records. This data included gender, age, body type, smoking history, drinking history, BMI, and basic medical history (hypertension, diabetes, etc.), as well as levels for the following indices: P2X3R, NGF, TrkA, urinary leukocyte, C-reactive protein, neutrophil count, erythrocyte sedimentation rate, and procalcitonin (PCT). The positive expression data of P2X3R, NGF, and TrkA were observed as brown particles stained light yellow to deep brown in the nucleus under a microscope.

**Fig. 1:**
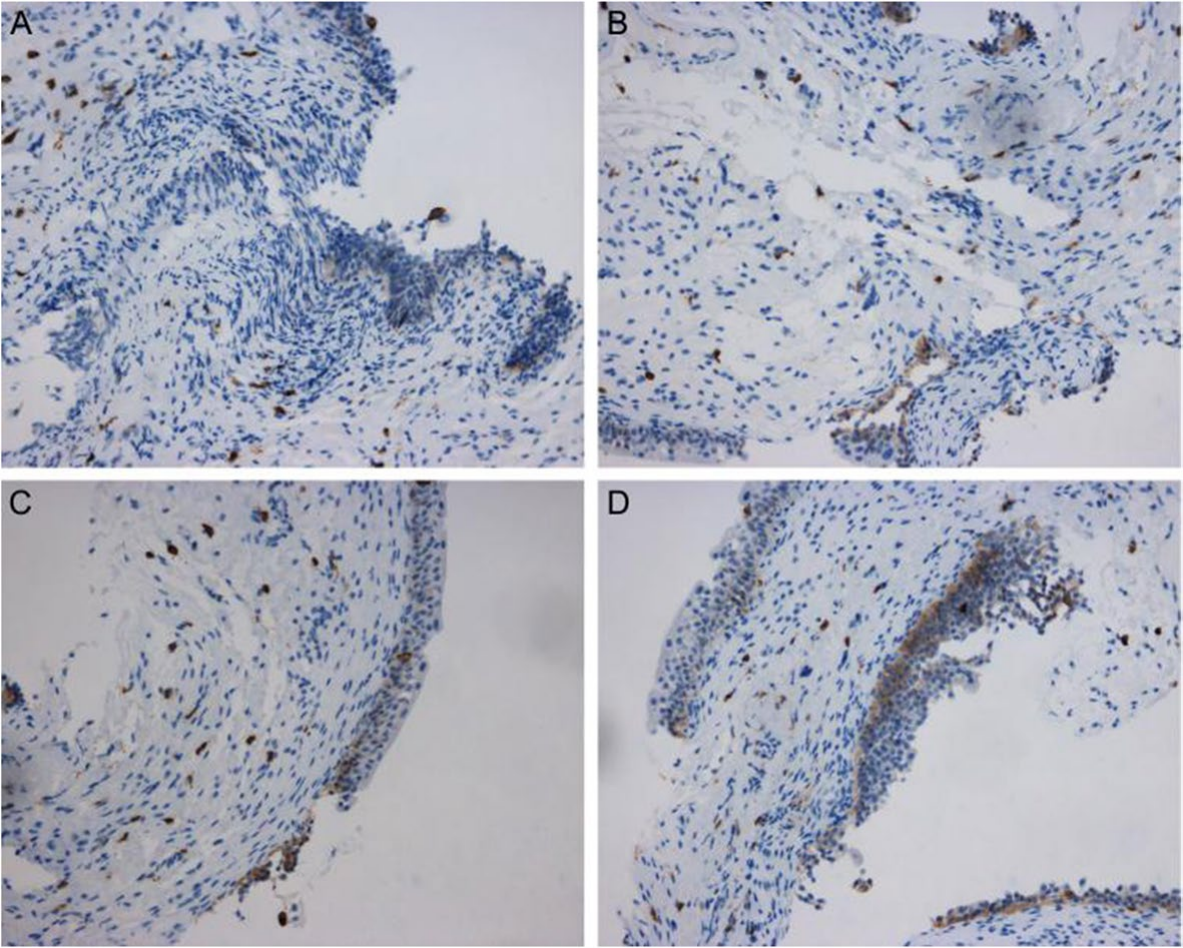
Immunohistochemical display of CD117 expression in interstitial cystitis

**Fig. 2:**
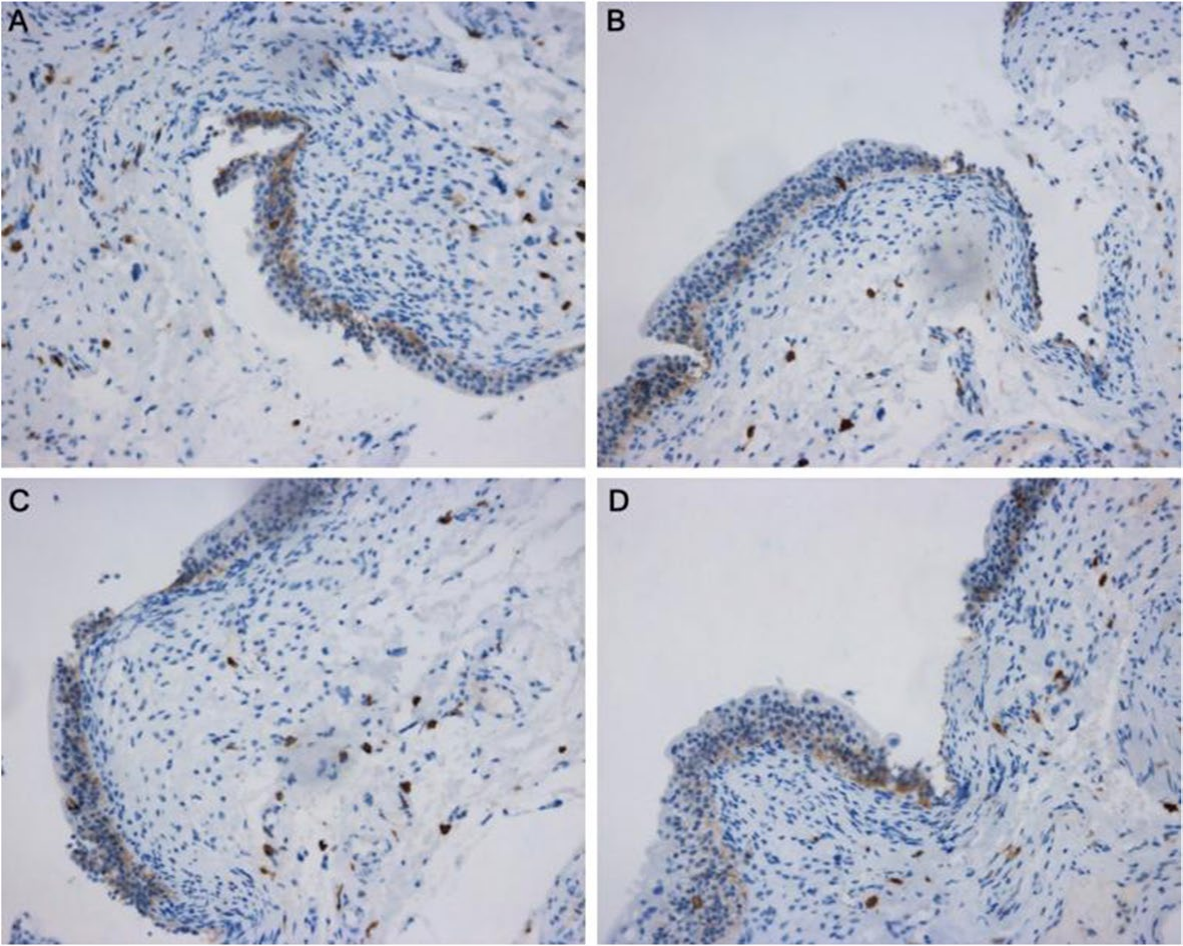
Immunohistochemical display of P2X3 receptor expression in interstitial cystitis

**Fig. 3:**
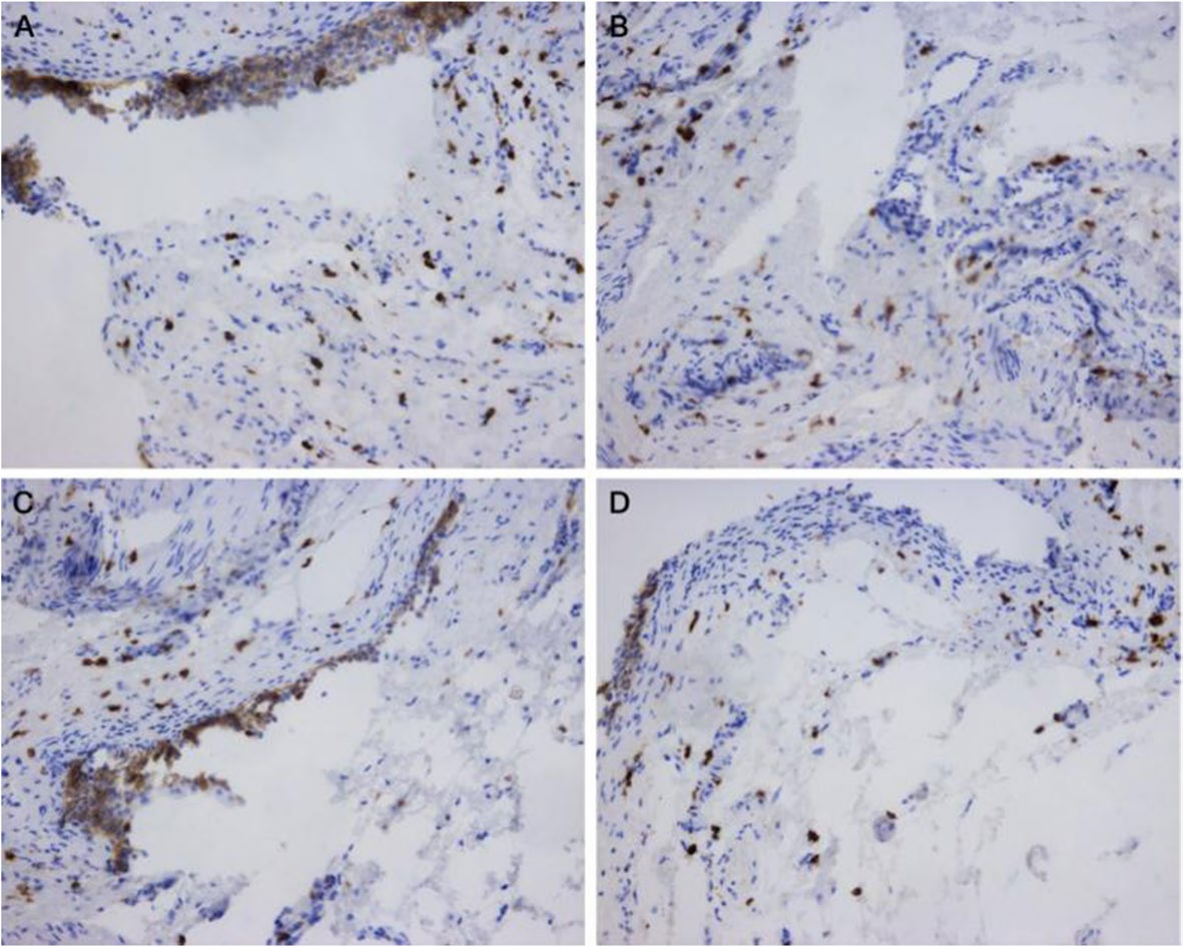
Immunohistochemical display of NGF expression in interstitial cystitis

**Fig. 4:**
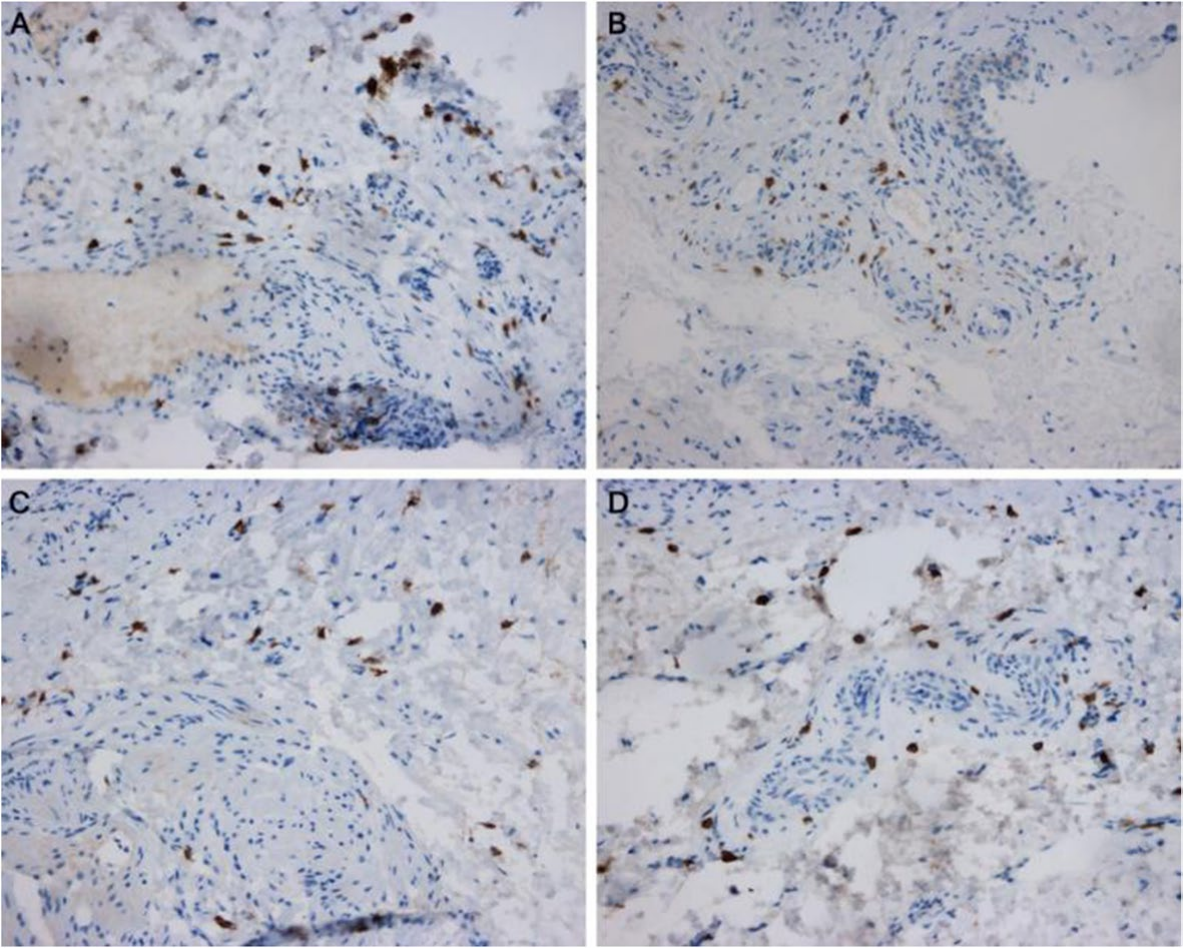
Immunohistochemical display of TrkA expression in interstitial cystitis

The scoring method for the proportion of positive cells was as follows: record 0 points if the proportion was less than 5%; record 1 point if it was between 6%-25%; record 2 points if it was between 26%-50%; record 3 points if it was between 51%-75%; record 4 points for values between 76%-100%. The scoring method based on color was as follows: 0 points for no color; 1 point for light brown/yellow; 2 points for deep brown/yellow; 3 points for brown/brown. The two scores were then multiplied together to obtain the final score. A score of 0–1 points was recorded as negative (-), 2–4 points as weakly positive ( +), 5–8 points as positive (+ +), and 9–12 points as strongly positive (+ + +) (See Figs. 1, 2, 3 and 4).

### Statistical methods

All statistical analyses were conducted using SPSS 26.0 (IBM, Armonk, NY, USA). Using SPSS, patients were divided into a model group and a validation group in a 7:3 ratio. Measurement data that followed a normal distribution were expressed as the mean ± standard deviation (x ± s) and compared between groups using the *t* test; non-normally distributed measurement data were expressed as median (interquartile range) [M (P25 ~ P75)], and intergroup comparisons were conducted using the Mann Whitney U test. Categorical data were compared between groups using the chi-square (χ2) test.

Spearman’s rank correlation analysis was employed to investigate the correlation between various risk factors and patient mortality, and logistic regression analysis was used to identify the key risk factors associated with worsening conditions in patients with BPS/IC. The results of these analyses formed the basis for the construction of the nomogram, the performance of which was then evaluated by calculating the AUC, or area under the receiver operating characteristic (ROC) curve. Additionally, the net clinical benefit and effectiveness of the nomogram were assessed using decision curve analysis (DCA). All statistical tests were two-tailed, and a *P*-value < 0.05 was considered statistically significant. Sort the importance of each variable using the random forest algorithm. *P* < 0.05 indicates a significant difference.

## Result

### Baseline characteristics

The study included a total of 1006 patients diagnosed with BPS/IC who visited the urology department of Shanxi Provincial People's Hospital. Among them, 704 patients were assigned to the model group, and the remaining 302 patients were assigned to the validation group. Table 1 presents a comparison between the baseline characteristics of the two groups, including data on age, BMI, smoking history, alcohol and coffee consumption, hypertension, diabetes, number of pregnancies and deliveries, menopause status, pelvic inflammation, overall bladder condition, anxiety, use of psychotropic drugs, presence of other types of cystitis, urinary leukocyte count, as well as CD117, P2X3R, NGF, TrkA, CRP, NLR, ESR, ALT, AST, Scr, and PCT levels. No statistically significant differences were observed between the two groups in terms of the resultant interstitial cystitis score, interstitial cystitis index, or questionnaire (*P* > 0.05).

**Table 1:** Clinical baseline characteristics of BPS/IC patients in the model and validation sets

	**Training group** **(*n* = 704)**	**Validation group** **(*n* = 302)**	***P* value**
Age, y	62.5 (56.0–65.0)	63.0 (57.0–66.0)	0.959
BMI	20.0 (17.5–23.0)	21.0 (18.0–22.0)	0.538
Smoking	33 (4.7)	17 (5.6)	0.529
Alcohol consumption	58 (8.2)	23 (7.6)	0.739
Coffee	24 (3.4)	18 (6.0)	0.064
Hypertension	67 (9.5)	33 (10.9)	0.493
Diabetes	361 (51.3)	151 (50.0)	0.710
Number of pregnancies(1/2/3)	657/41/6	290/8/4	0.082
Number of delivery(0/1/2/3)	5/669/28/2	5/289/6/2	0.161
Pausimenia	701 (99.6)	301 (99.7)	0.826
Pelvic inflammation	376 (53.4)	152 (50.3)	0.370
Overactive bladder	661 (93.9)	284 (94.0)	0.928
Anxiety	418 (59.4)	164 (54.3)	0.136
Taking psychotropic drugs	397 (56.4)	153 (50.7)	0.094
Other types of cystitis	15 (2.1)	12 (4.0)	0.097
CD117	6.0 (9.0–12.0)	6.0 (9.0–12.0)	0.668
P2X3R	4.0 (9.0–12.0)	4.0 (9.0–12.0)	0.293
NGF	6.0 (9.0–12.0)	6.0 (9.0–9.0)	0.351
TrkA	6.0 (6.0–8.0)	6.0 (6.0–8.0)	0.821
Urinary leukocyte	4 (0.6)	1 (0.3)	0.624
CRP, mg/L	5.5 (4.6–6.4)	5.5 (5.1–6.4)	0.112
NLR	2.4 (1.9–2.6)	2.4 (1.9–2.6)	0.605
ESR, mm/1 h	7.0 (5.0–8.0)	7.0 (5.0–8.0)	0.920
ALT, U/L	35.0 (25.0–42.0)	31.5 (25.0–39.0)	0.152
AST, U/L	26.0 (24.0–35.0)	34.0 (24.0–35.0)	0.157
Scr, µmol/L	67.0 (58.0–72.0)	67.0 (58.0–72.0)	0.257
Procalcitonin,ug/L	0.4 (0.3–0.5)	0.4 (0.3–0.5)	0.339
Interstitial cystitis score	17.0 (16.0–18.0)	17.0 (16.0–18.0)	0.685
Interstitial cystitis index	14.0 (13.0–15.0)	14.0 (13.0–15.0)	0.755
Questionnaire	19.0 (17.0–21.0)	19.0 (17.0–21.0)	0.065
Interstitial cystitis score (at 3 monthes)	15.0 (5.0–17.0)	15.0 (5.0–17.0)	0.687
Interstitial cystitis index (at 3 monthes)	12.0 (5.0–14.0)	12.0 (5.0–14.0)	0.393
Questionnaire (at 3 monthes)	16.0 (9.0–18.0)	15.0 (9.0–18.0)	0.182
Interstitial cystitis score (at 6 monthes)	15.0 (4.0–17.0)	15.0 (4.0–16.0)	0.324
Interstitial cystitis index (at 6 monthes)	12.0 (4.0–14.0)	11.0 (4.0–14.0)	0.306
Questionnaire (at 6 monthes)	16.0 (4.0–19.0)	15.0 (4.0–18.0)	0.219
Interstitial cystitis score (at 12 monthes)	15.0 (6.0–17.0)	15.0 (7.0–17.0)	0.156
Interstitial cystitis index (at 12 monthes)	12.0 (5.0–14.0)	12.0 (6.0–14.0)	0.310
Questionnaire (at 12 monthes)	16.0 (9.0–18.0)	15.0 (9.0–18.0)	0.204

### Logistic regression analysis of risk factors affecting prognosis in patients with interstitial cystitis

Univariate logistic regression analysis was performed to assess the correlation between various risk factors and prognosis. The results indicated that age, diabetes, pelvic inflammation, and the expression of CD117, P2X3R, NGF, TrkA, ESR, and PCT were significantly correlated with IC prognosis (all *P* < 0.05). These factors were therefor included in the multivariate analysis.

The results of multivariate logistic regression analysis showed that age (HR = 1.096, 95% CI:1.019–1.112; *P* < 0.006), CD117 (HR = 1.515, 95% CI:1.295–1.772; *P* < 0.001), P2X3R (HP = 2.176, 95% CI:1.881–2.514; *P* < 0.001), NGF (HR = 1.765, 95% CI:1.476–2.110; *P* < 0.001), and TrkA (HR = 2.077, 95% CI:1.679–2.571; *P* < 0.001) were all independent risk factors associated with poor prognosis in patients with IC (*P* < 0.05) (see Table 2).

**Table 2:** Univariate and multivariate logistic regression analyses for prognosis of patients in the model group

Variables	Univariate analysis		Multivariate analysis	
	**HR (95% CI)**	***P*-value**	**HR (95% CI)**	***P*-value**
Age, y	1.006 (1.043,1.089)	< 0.001	1.096 (1.019,1.1121)	0.006
Smoking	0.339 (0.183,0.628)	0.001		
Alcohol consumption	0.608(0.385,0.962)	0.034		
Coffee	0.612 (0.328,1.142)	0.123		
BMI	0.713 (0.465,1.274)	0.416		
Hypertension	0.917 (0.553,1.519)	0.736		
Diabetes	3.744 (2.204,4.781)	< 0.001		
Pelvic inflammation	2.799 (1.023,3.319)	< 0.001		
Overactive bladder	0.713 (0.386,1.382)	0.334		
CD117	1.448 (1.355,1.546)	< 0.001	1.515 (1.295,1.772)	< 0.001
P2X3R	2.331 (2.090,2.599)	< 0.001	2.176 (1.881,2.514)	< 0.001
NGF	1.636 (1.508,1.774)	< 0.001	1.765 (1.476,2.110)	< 0.001
TrkA	2.146 (1.896,2.429)	< 0.001	2.077 (1.679,2.571)	< 0.001
CRP, mg/L	1.217 (1.041,1.422)	0.014		
NLR	1.038 (0.756,1.424)	0.819		
ESR, mm/1 h	1.114 (1.082,1.210)	< 0.001		
Procalcitonin,ug/L	0.154 (0.050,0.474)	0.001		

### Constructing the nomogram

**Fig. 5:**
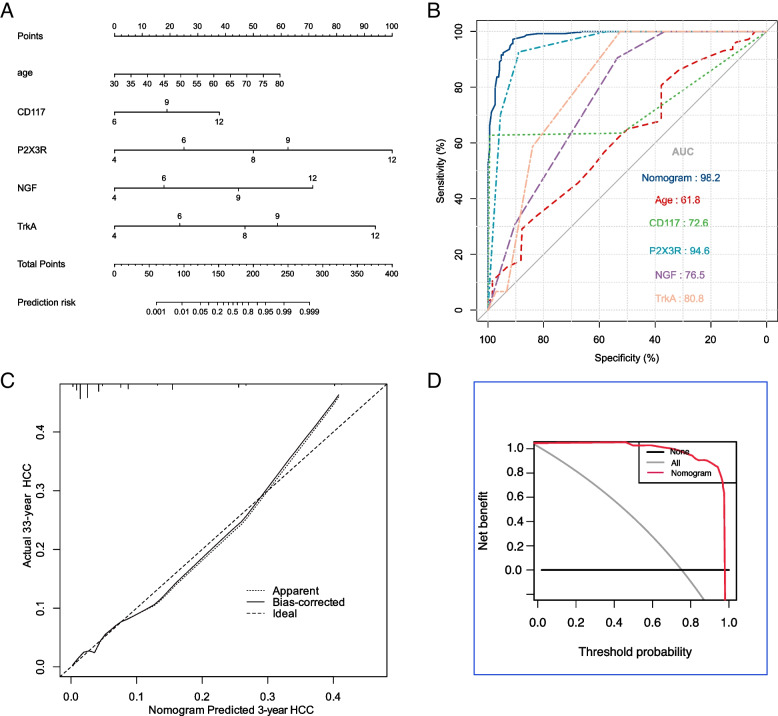
**A** Column Chart Model. **B **ROC curve. **C **Calibration curve. **D **Decision curve

Using R 4.1.1 software, a functional model was constructed by incorporating the factors that were found to affect prognosis by the multivariate logistic regression analysis. This was used to develop and plot a nomogram. The scores for each number and category related to the aforementioned factors were aggregated to obtain the total score. By drawing a downward straight line, one arrives at the intersection point with the prognosis coordinate axis, representing the patient’s estimated survival time. This can also be used to predict mortality probability at any given point in time (Fig. 5A). CD117, P2X3R, NGF, and TrkA were all identified as important factors in predicting the prognoses of patients with IC (see Fig. 5).

The results of the ROC curve analysis demonstrated that the AUC and 95% confidence interval for nomogram, age, CD117, P2X3R, NGF, and TrkA were 0.982(0.960–0.100), 0.618(0.552–0.685), 0.726(0.633–0.818), 0.946(0.893–0.999), 0.765(0.690–0.840), and 0.808(0.757–0.859), respectively (see Fig. 5B).The randomly selected data were divided into training and validation sets in a 7:3 ratio, and the ROC curve was drawn using R 4.1.1 software.

### Effectiveness evaluation of the column chart model

The performance of the nomogram was evaluated using the AUC, calibration curve, and decision curve. Additionally, the results of a consistency analysis showed that the overall survival rate curve in the nomogram closely aligned with the 45° diagonal in the calibration chart, indicating a high degree of consistency between the model and the actual data (Fig. 5C). Furthermore, decision curve analysis demonstrated that the model provides useful clinical benefits (Fig. 5D).

### Verification of the column chart

**Fig. 6:**
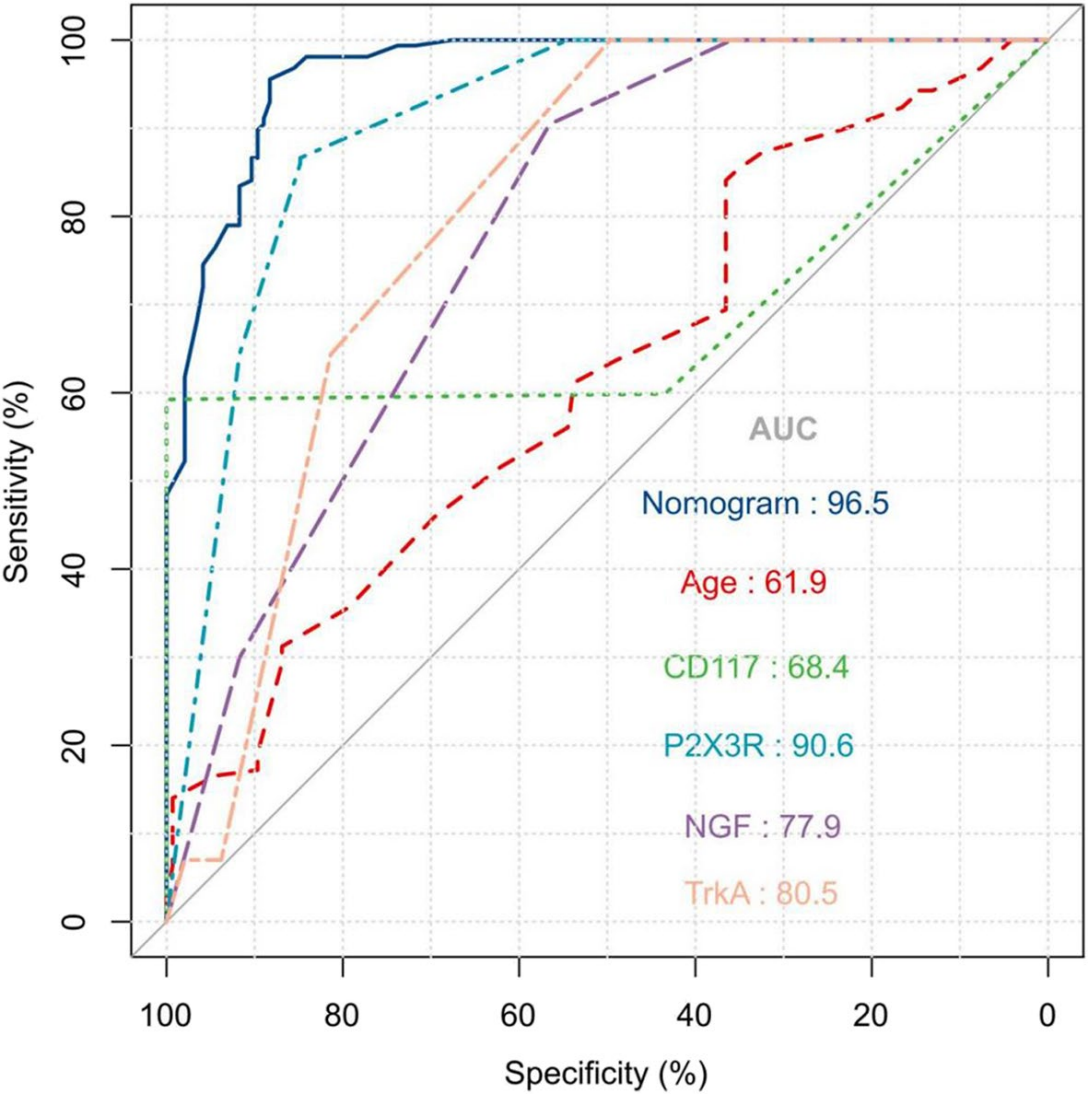
Verification of the ROC curve

To validate the column chart model as a useful tool for predicting the prognosis of BPS/IC, we conducted an analysis using the validation cohort consisting of 302 people. ROC curve analysis showed that the model exhibited considerable discriminative ability. The AUC values obtained were 0.965(0.932–0.100), 0.619(0.550–0.687), 0.684(0.604–0.764), 0.906(0.855–0.957), 0.779(0.706–0.852), and 0.805(0.744–0.866), for nomogram, age, CD117, P2X3R, NGF, and TrkA, respectively, indicating that the presence of these factors offers clinical value as prognostic indicators (Fig. 6).The above genes are various genes with increased expression levels in interstitial cystitis tissue immunohistochemistry, and their amounts can be used to evaluate the severity of interstitial cystitis and judge the treatment effect.

### Building a random forest model

Using R4.2.3 system, 1006 samples were divided into training sample set (*n* = 704, accounting for 70%) and testing sample set (*n* = 302, accounting for 30%) using simple random sampling method. The importance of each variable in the training sample set was ranked based on the random forest algorithm.

The training sample set uses the random forest algorithm to construct a random forest model based on independent risk factors (age, CD117, P2X3R, NGF, TrkA) obtained from logistic regression analysis. ntree is 20, and variables mtry in each tree is 1. We evaluate the classification performance of the model by calculating the confusion matrix between the training and testing sets, and then analyze the calibration curve and receiver operating characteristic curve. Finally, in order to compare whether the AUC differences between different variables are statistically significant, Delong tests were performed on each variable in the training and testing sets.

### Analysis of random forest results and model validation

#### Result analysis

**Fig. 7:**
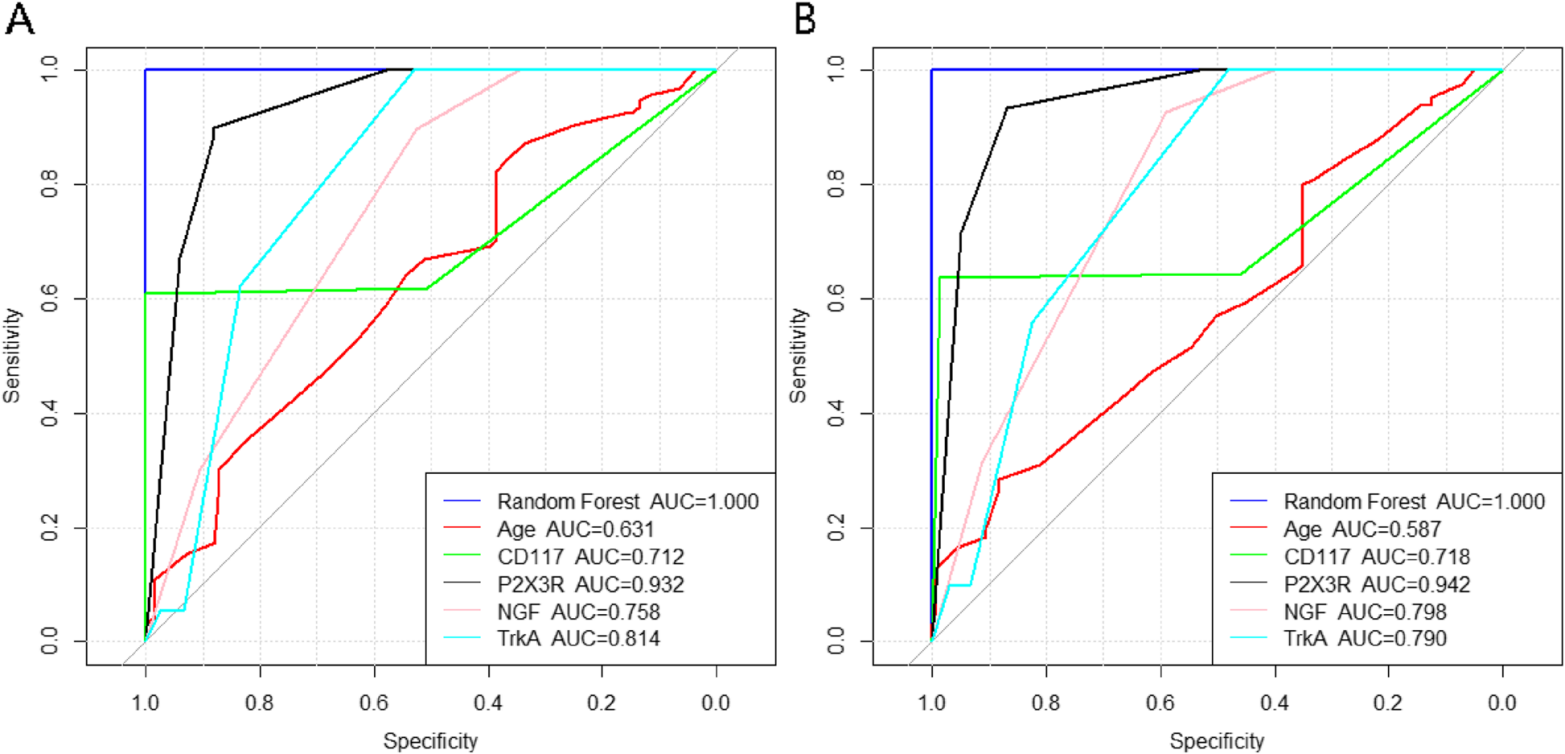
ROC curves for the training and testing sets of a random forest. **A** Training set ROC; **B** Test set ROC

The number of training samples is 704, and the test set data is classified based on the above parameters. Based on the contribution rate of the decision tree to obtain feature importance, the results showed that the AUC of the random forest was 1.000, the AUC of age was 0.631, the AUC of CD117 was 0.712, the AUC of P2X3R was 0.932, the AUC of NGF was 0.758, and the AUC of TrkA was 0.814 (Fig. 7A). The Delong test results showed that there were statistically significant differences in AUC among age, CD117, P2X3R, NGF, and TrkA (Tables 3 and 4).

**Table 3:** Test set Delong test

	Z	P
Age	13	< 2e-16
CD117	9.4	< 2e-16
P2X3R	4.3	= 2e-05
NGF	8.3	< 2e-16
TRKA	8.1	= 5e-16

**Table 4:** Training set Delong test

	Z	P
Age	18	< 2e-16
CD117	15	< 2e-16
P2X3R	7.1	= 2e-12
NGF	14	< 2e-16
TRKA	11	< 2e-16

#### Model validation

The random forest algorithm was used to predict the risk prediction model constructed on the training set. The results showed that the AUC of the random forest was 1.000, the AUC of age was 0.587, the AUC of CD117 was 0.718, the AUC of P2X3R was 0.942, the AUC of NGF was 0.798, and the AUC of TrkA was 0.790 (Fig. 7B), as can be seen from Table 5, The use of random forest models to predict the prognosis of patients with interstitial cystitis after bladder water dilation surgery has high accuracy.

**Table 5:** Precision of Training Set and Precision of Test Set

Training set accuracy	Test set accuracy
Accuracy: 0.999	Accuracy: 0.997
95% CI: (0.992, 1)	95% CI: (0.982, 1)
No Information Rate: 0.547	No Information Rate: 0.55
*P*-Value [Acc > NIR]: < 2e-16	*P*-Value [Acc > NIR]: < 2e-16
Kappa: 0.997	Kappa: 0.993
Mcnemar's Test P-Value: 1	Mcnemar's Test P-Value: 1
Sensitivity: 1.000	Sensitivity: 1.000
Specificity: 0.997	Specificity: 0.994
Pos Pred Value: 0.997	Pos Pred Value: 0.993
Neg Pred Value: 1.000	Neg Pred Value: 1.000
Prevalence: 0.453	Prevalence: 0.450
Detection Rate: 0.453	Detection Rate: 0.450
Detection Prevalence: 0.455	Detection Prevalence: 0.454
Balanced Accuracy: 0.999	Balanced Accuracy: 0.997

#### Model calibration curve

The S-shaped calibration curve indicates a good consistency between the predicted probability of the model and the actual observed values. When the predicted probability approaches 0, the actual proportion of observed events also approaches 0; When the predicted probability approaches 1, the actual proportion of observed events also approaches 1. In this case, the model has good predictive ability and can accurately reflect the probability of events occurring.

**Fig. 8:**
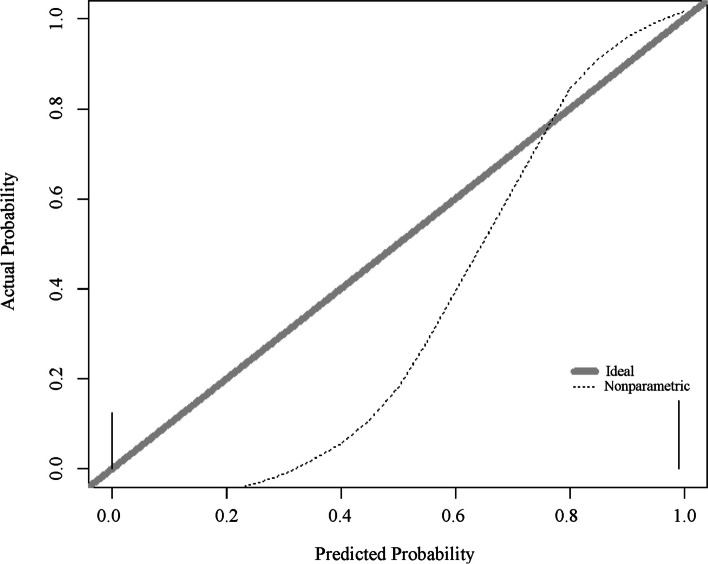
Calibration curve of random forest model

By observing the calibration curve, we can draw the following conclusion: if the calibration curve is close to the ideal diagonal, it indicates that the predicted probability of the model is highly consistent with the actual observed values, and the model has good calibration performance (Fig. 8)*.*

## Discussion

While the etiology and pathogenesis of BPS/IC has not yet been fully elucidated, current understanding encompasses several key aspects. Firstly, epithelial damage and reduced permeability after infection have been implicated [6]. Secondly, an increase in mast cell numbers and elevated levels of acellular cells, IgE, IgG, and estrogen often lead to the production of several inflammatory mediators such as substance P and histamine, contributing to inflammation and aggravation [7]. Thirdly, increases in the production of bladder mucosal epithelial growth factor (APF) have been linked to the pathogenesis of BPS/IC. APF’s interaction with CKAP4/P63 receptors on bladder epithelial cell membranes has been shown to impact the growth of the cells’ epithelial layer, leading to thinning and increased permeability of the mucosal epithelium. When this process occurs, potassium molecules and other harmful elements in urine may leak into interstitial cells, resulting in inflammation and pain [8]. Fourthly, glomerular punctate hemorrhage, a characteristic feature of IC, is believed to be caused by obesity-induced overexpression of inflammatory mediators, capillary endothelial growth factor, hypoxia-inducible factor, and tumor necrosis factor, leading to an increase in the surface layer of interstitial capillaries in the bladder. Increased concentrations of blood fibrinolytic enzymes further contribute to small capillary exudation [9]. Lastly, mast cell and immune cell activation, along with the release of inflammatory mediators such as histamine, substance P, nerve growth factor (NGF), and estrogen, can stimulate the demyelinating nerve fibers of the bladder, resulting in bladder pain syndrome [10]. The clinical manifestations of BPS/IC often include persistent pelvic pain, which seem to be influenced by specific genes such as CD117, P2X3R, NGF, and TrkA.

This study analyzed the independent risk factors affecting the prognosis of patients with BPS/IC after bladder water dilation surgery, relying on general preoperative information and various biomarkers (CD117, P2X3R, NGF, and TrkA) plus clinical pathological features for the basis of the investigation. This foundation was used to develop a predictive column chart model in the form of a nomogram. The results showed that older age, as well as elevated levels of CD117, P2X3R, NGF, and TrkA were associated with poor prognoses in patients with BPS/IC following water dilation surgery.

Increased mast cell expression in patients with BPS/IC has been observed in previous research and suggests that mast cells play a key role in inflammatory reactions as well as the development, persistence, and pain associated with BPS/IC [11]. The bladders of BPS/IC patients exhibit significant increases in mast cell numbers, underscoring their importance in the pathophysiology of the condition. Activation of mast cells can be triggered by various factors, including neuropeptides (e.g., substance P and neurotensin), NGF, tumor necrosis factor-α, and stem cell factor (SCF), which can cause mast cells to release inflammatory mediators.

Researchers have proposed the theory of neurogenic inflammation, which suggests that neurogenic inflammation occurs when afferent neurons release inflammatory mediators, leading to the stimulation and sensitization of activated inflammatory cells (such as mast cells and leukocytes). These, in turn, release more inflammatory mediators, forming a feedback cycle [12]. Our immunohistochemical analysis using CD117 as a marker confirmed an increased number of mast cells in patients diagnosed with BPS/IC.

P2X receptors, belonging to the ligand-gated ion channel family, are ion channels that selectively conduct cations. Molecular cloning has confirmed that P2X has seven subunits, referred to as P2X1 ~ P2X7. Among these, P2X3 receptors have been found to be closely correlated with pain transmission in both the peripheral and central nervous systems, which play important roles in the development and persistence of pain in the sensory nervous system [13]. In addition, NGF activates TrkA, which is expressed in various organs and tissues.The binding of NGF to TrkA on protein membrane triggers the activation of tyrosine kinase, and can lead to the production of phosphorylated tyrosine in amino acid residues.In the process of inactivation, the activation loop of TrkA inserts into the center of the enzyme’s activation site, blocking the entry site of adenosine triphosphate (ATP) and inhibiting tyrosine kinase activity. However, when NGF dimer binds to TrkA dimer, the activation loop is released, allowing TrkA to utilize ATP to self-phosphorylate (pY) on the tyrosine residues (Y676, Y680, Y681) located on the opposite activation loop [14]. This activated form of TrkA then phosphorylates cell matrix proteins and transmits information to the nucleus through the NGF/TrkA signaling pathway. NGF, which acts on peripheral nociceptive neuronal endings, first fuses with TrkA on the cell membrane before being absorbed by the cell body. It is then transmitted through axons to the cell body of the dorsal root ganglion. The activation of the downstream intracellular signal transduction system leads to the production of various proteins, ultimately contributing to the sensation of pain [15]. NGF’s role in pain occurs through two different processes. The first occurs during the fetal period, when NGF is involved in the growth of nerve fibers that transmit pain sensations. Later in adulthood, NGF also plays a role in inducing pain [16].

In this study, univariate logistic regression analysis revealed significant correlations between various factors and the prognosis of IC. These factors included age (HR = 1.096, 95% CI:1.019–1.112; *P* < 0.006), CD117 (HR = 1.515, 95% CI:1.295–1.772; *P* < 0.001), P2X3R (HP = 2.176, 95% CI:1.881–2.514; *P* < 0.001), NGF (HR = 1.765, 95% CI:1.476–2.110; *P* < 0.001), and TrkA (HR = 2.077, 95% CI:1.679–2.571; *P* < 0.001). Therefore, all of these may be considered independent risk factors that predict poor prognosis in patients with IC.

Column charts serve as valuable tools for quantifying and predicting the probability of a clinical event occurring, enabling clinicians to make informed decisions and stratify risks. This study employed logistic regression analysis to identify independent factors affecting the prognosis of patients with BPS/IC after hydrodilation surgery. Subsequently, a nomogram based on these factors was constructed, and its performance was evaluated through consistency analysis, among other analytic methods. The results showed that the nomogram exhibited strong predictive capabilities. The model was further validated using metrics such as the AUC, calibration curve, and decision curve. Consistency analysis revealed a close alignment between the overall survival rate curve in the nomogram and the 45° diagonal in the calibration chart, indicating that the model has a high level of consistency. Moreover, decision curve analysis confirmed the model’s clinical potential. These findings attest to the robustness and clinical applicability of the nomogram. Its validation reinforces its value as a tool that can provide personalized risk assessments and guide the adjustment of clinical plans for patients with BPS/IC after hydrodilation surgery.

In conclusion, this study identified increased age, CD117 expression, P2X3R expression, NGF expression, and TrkA expression as independent risk factors associated with poor short-term prognosis in patients with interstitial cystitis. The column chart model that was developed based on these factors demonstrated its predictive value identifying individuals at risk of poor short-term prognosis in this patient population. Moreover, this model also takes into account pathological immunohistochemical scores, laboratory indicators, and symptomatology indicators, providing clinicians with a comprehensive approach that is both convenient and user-friendly. Specifically, it facilitates the selection of appropriate diagnostic and treatment methods based on an individual’s condition, ultimately improving prognosis. In addition, we found that the random forest model has better predictive ability and can understand the importance of various features. The sensitivity, specificity, and diagnostic accuracy of this model are higher than traditional logistic regression models, and with the increase of features and sample size, the diagnostic efficiency and generalization ability of this model will be further improved. The establishment of this model helps to achieve prognostic risk management for patients with interstitial cystitis and more efficiently optimize the allocation of medical resources.

However, it is important to acknowledge the limitations of the study. Notably, it was conducted at a single center with a relatively small sample size and lacked external verification. Therefore, further research involving multiple centers and larger sample sizes is needed to further validate the effectiveness of the column chart model.In the further work, we will use more biomedical based deep learning methods, including decision tree algorithm [17], support vector machine (SVM) algorithm [18], naive Bayesian algorithm [19], Xgboost algorithm [20], principal component analysis PCA algorithm [21], DBSCAN algorithm [22], etc., to improve the performance of the constructed model.

## Data Availability

Data available,A total of 1006 patients who were diagnosed with BPS/IC and received treatment in shanxi provincial people’s hospital outpatient department between June 2012 and June 2022 were selected as the research subjects.The datasets generated and/or analysed during the current study are not publicly available but are available from the corresponding author on reasonable request. (shuangweibing@126.com).
